# Characterizing the 16p12.2 Microdeletion and Its Association with Psychotic Disorders: A Genetic and Clinical Perspective

**DOI:** 10.1192/j.eurpsy.2025.1136

**Published:** 2025-08-26

**Authors:** R. González Núñez, L. Mallol Castaño, R. Albillos Pérez, A. Castelao Escobar, C. Alguacil Núñez, P. Sanz Sánchez

**Affiliations:** 1 Hospital Universitario Puerta de Hierro, Madrid, Spain

## Abstract

**Introduction:**

This report presents a case involving a patient diagnosed with a 16p12.2 microdeletion and associated psychotic symptoms.

**Objectives:**

Throughout the course of this case, we will develop a deeper understanding of the symptoms associated with this chromosomal anomaly.

**Methods:**

The case is described below: A 15-year-old woman was admitted to the psychiatric unit at Puerta de Hierro Hospital due to behavioral disturbances. The patient was born with plagiocephaly, experienced learning difficulties, and has a documented history of a 16p12.2 microdeletion, epilepsy, and obesity. Her first contact with mental health services occurred in 2017 due to disruptive behaviors, and she began treatment with risperidone. Her condition worsened in 2020, leading to three hospital admissions and treatment changes, including aripiprazole, clozapine, and paliperidone, under the diagnosis of an unspecified psychotic disorder related to the 16p12.2 microdeletion. Currently, she is on sertraline (200 mg), paliperidone (12 mg), and valproic acid (600 mg).

The patient lives with her parents and twin sister, who has the 16p12.2 microdeletion and schizophrenia. Recently, she has shown increased irritability, heteroaggressiveness, insomnia, and difficulties with emotional regulation. The diagnosis is behavioral disturbances in the context of the 16p12.2 microdeletion.

**Results:**

Upon admission, the valproic acid dosage was increased to 900 mg, and olanzapine 2.5 mg was introduced. This was accompanied by therapy and a structured environment. The result was progressively more syntonic behavior and an improved capacity for self-regulation.

**Conclusions:**

Most genomic disorders result from non-allelic homologous recombination (NAHR) between segmental duplications. The clinical presentation of the 16p12.2 microdeletion is highly heterogeneous and includes developmental and growth delays, craniofacial anomalies, epilepsy, sleep disorders, learning difficulties, hypotonia, cardiac malformations, and psychiatric and behavioral disturbances. Diagnosis is established through chromosomal microarray analysis or other genomic tests. Treatment is directed at addressing the specific problems identified.

**Disclosure of Interest:**

None Declared

